# Can Clinicians Start Prescribing Inclisiran for Hypercholesterolemia Today? A Review of Clinical Studies for Internal Medicine Physicians and Endocrinologists

**DOI:** 10.7759/cureus.16664

**Published:** 2021-07-27

**Authors:** Abdullah Jahangir, Syeda Sahra, Michael Krzyzak

**Affiliations:** 1 Internal Medicine, Northwell Health, Staten Island, USA

**Keywords:** inclisiran, pcsk9 inhibitors, aln-pcs

## Abstract

The safety profile and efficacy margin of inclisiran as a lipid-lowering drug have been assessed in clinical trials and are underway in subgroups with relevant co-morbidities. This systematic review looks at the clinical trials that have been conducted to comment on its safety and efficacy. The conclusions can serve as a guide for practicing physicians and researchers for following current and future cohorts of patients. PubMed, Cochrane, Embase, Scopus, CINAHL, Web of Science, and Clinicaltrials.gov were searched comprehensively using the terms “Inclisiran”, “ALN-PCSsc”, and “ALN-PCS” using the Boolean operator “OR” with data cut-off date of June 28, 2020. The outcomes of safety and efficacy were collected and charted for the systematic review. In our study, eight clinical trials were included in the final study: the ORION (1,2,7,9-11) trials and two clinical trials (phase 1 randomized clinical trials) done before ORION trials. Favourable efficacy in terms of LDL levels and PSCK9 levels was observed across all eight clinical trials. No severe adverse effects, safety concerns, or fatalities attributable directly to inclisiran were reported. Therefore, our study results suggest a positive efficacy and safety profile of inclisiran as a lipid-lowering drug in clinical trials.

## Introduction and background

Proprotein convertase subtilisin/kexin type 9 (PCSK9) is encoded by the PCSK9 gene, and it regulates the hepatic low-density lipoprotein (LDL) receptors. It is secreted in the liver and modulates the activity of LDL receptors. PCSK9 binds the LDL receptors in their active form, leading to their lysosomal destruction [1, 2]. Once PCSK9 levels were linked with hypercholesterolemia, there have been many trials to assess different agents which block this interaction between PCSK9 and LDL receptors. Various preclinical studies linked the lower level of circulating PCSK9 with decreased serum cholesterol levels. In humans, most of the initial trials involving PCSK9 inhibition used monoclonal antibodies like evolocumab and alirocumab directed against PSCK9 that bind this enzyme and cause its degradation.

However, the advent of small interfering RNA (siRNA) opened up a new avenue of genetic manipulation by silencing particular genes [3]. siRNA is a double-stranded RNA molecule that degrades mRNA sequences after transcription, hence preventing mRNA translation, ultimately leading to a decline in the production of the enzymes coded by those genes [4]. For PCSK9, one such drug, inclisiran, is being evaluated in a series of clinical trials-a small interfering RNA that blocks the production of hepatic PCSK9.

Many trials have been undertaken to calculate the safety profile and efficacy of inclisiran in a wide array of populations. The ORION trials are specifically designed to assess treatment outcomes of inclisiran in the broader population [5]. Some of the trials are still underway. Recently, initial results from the primary phase 3 clinical trials have been reported. More extensive trials with more participants with co-morbidities are underway. In this systematic review, we will look at the safety and efficacy outcomes reported in all the clinical trials involving inclisiran to better understand its associated clinical outcomes.

## Review

Literature search

A broad literature search was done on PubMed, Cochrane, Embase, Scopus, CINAHL, Web of Science, and Clinicaltrials.gov using the terms “Inclisiran,” “ALN-PCSsc,” and “ALN-PCS.” These terms were combined using the Boolean operator “OR.” The search was not limited to any region or any language if the English translation was available. The data cut-off date for this search was June 28, 2020. Relevant articles from recent conferences were also included.

Eligibility criteria

Only those studies that met the following criteria were included in the review: (a) inclisiran was the primary intervention drug; (b) original clinical studies with the human population; (c) safety and efficacy outcomes were reported. 

Study selection

For the study, relevant studies were reviewed based on the title and abstract. The potential articles obtained from the first review were then screened again with full text by the same reviewers. Conference abstracts and presentations reporting the interim results of the ongoing inclisiran trials were also included.

Data extraction

**Figure 1:**
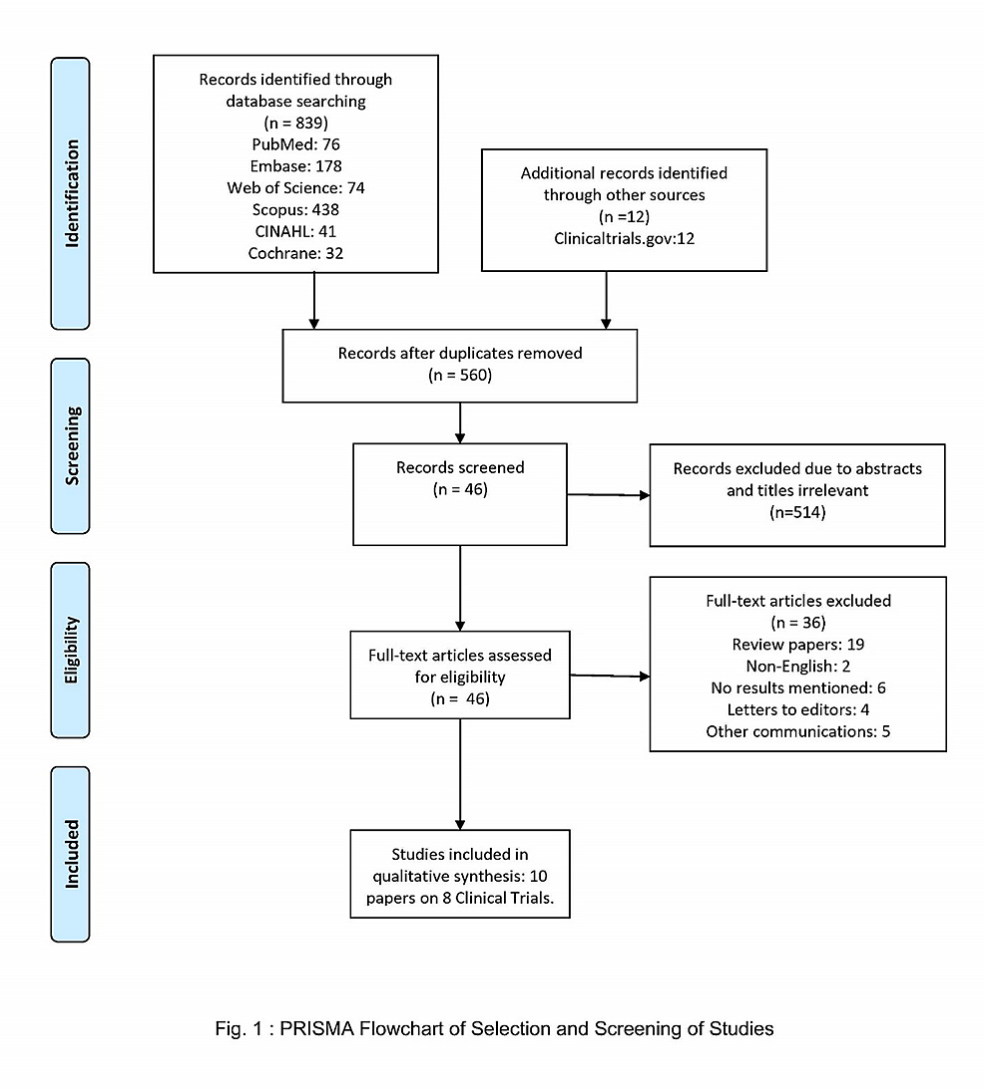
PRISMA diagram for screening of clinical studies.

Data extraction was done using pre-specified tables of baseline characteristics, safety, and efficacy. The parameters recorded were author, year, patient population, age, regimen, baseline BMI, baseline LDL cholesterol, and any changes in LDL-cholesterol, PCSK9, and adverse events. The data was recorded as mean, medians, and percentages. The data is demonstrated in Tables 1-3. The PRISMA diagram for the screening of studies is attached (Figure 1).

**Table 1:** Baseline characteristics of participants involved in review

Author, Year	Trial # (Trial Name)	Phase	Study Design	Administered Drugs and Dosages	Number of Participants	Dosing Schedule Of Intervention Drug	Median or Mean Age (Years)	Mean Baseline BMI (kg/m2)	Mean Baseline LDL-C	Mean Baseline PCSK9	Previous Diagnosis
Fitzgerald et al., 2014 [6]	NCT01437059	1	Randomised, single-blind, placebo-controlled trial	Inclisiran (from 0.015 up to 0.400 mg/kg)	24	One-hour infusion only once	51	28.7	3.7 mmol/L	1014.7 ng/ml	None
				Placebo	8		41.5	28.9	3.9 mmol/L	1067 ng/ml	
Fitzgerald et al., 2016 [7]	NCT02314442	1	Randomised, single-blind, placebo-controlled study in two stages	Single dose inclisiran from 25 mg up to 800 mg	18	Single dose	48	25.7	163 mg/dL	275.4 ug/L	None
				Placebo	6		46	25	131.5 mg/dL	279 ug/L	
				Multiple dose inclisiran with statins	9	Multiple doses of inclisiran plus statins (from 125 to 500 mg) ranging from one per week to once per month, or matching placebo plus statin	54	26.5	143.4 mg/dL	451 ug/L	
				Placebo with statin	4		58	26.3	143.1 mg/dL	460.7 ug/L	
				Multiple-dose inclisiran without statins	24	Multiple doses inclisiran or matching placebo (from 125 to 500 mg) ranging from one per week to once per month	51	25.5	139.3 mg/dL	317 ug/L	
				Placebo without statin	8		51	26.5	131.5 mg/dL	276.2 ug/L	
Fitzgerald et al., 2017 [7,8]	NCT02597127 (ORION-1)	2	Multicenter, double-blind, placebo-controlled, multiple ascending-dose trial	Single dose inclisiran	183	Single-dose patients had 200 to 500 mg of drug on Day 1, while two doses cohort had 100 to 300 mg on Day 1 and 90	63.3	28.1	125.8 mg/dL	428.6 ng/ml	None
				Single-dose placebo	63		62	30.1	128.5 mg/dL	404.7 ng/ml	
				Two doses of inclisiran	178		63.9	29.6	132.8 mg/dL	415.9 ng/ml	
				Two doses of placebo	60		62.8	29.2	125.2 mg/dL	431.3 ng/ml	
Raal et al., 2019 [9]	NCT02963311 (ORION-2)	2	Single-arm, open label, multicenter, pilot study	Single dose of inclisiran	4	300 mg of inclisiran on Day 1	57	N/A	Patient 1: 14.0 mmol/L Patient 2: 14.2 mmol/L Patient 3: 15.9 mmol/L Patient 4: 4.9 mmol/L	N/A	Familial hypercholesterolemia
Wright et al., 2020 [10]	NCT03159416 (ORION-7)	1	Phase 1, single-dose, open-label study	Single dose of inclisiran	23	300 mg inclisiran sodium on Day 1	57.4	30	115.5 mg/dL	268.73 ng/mL	Mild, moderate, or severe renal impairment
					8		52.1	27.63	135.7 mg/dL	336.5 ng/mL	Normal renal function
Raal et al., 2020 [11]	NCT03397121 (ORION-9)	3	Double-blind, randomized, placebo-controlled trial	Subcutaneous inclisiran with statins or ezetimibe	242	300 mg of subcutaneous injection of inclisiran or matching placebo on Days 1, 90, 270, and 450	56	N/A	151.4 mg/dL	452.2 ug/L	Heterozygous familial hypercholesterolemia
				Placebo with statins or ezetimibe	240		56	N/A	154.7 mg/dL	429.1 ug/L	
Ray et al., 2020 [12]	NCT03399370 (ORION-10)	3	Randomized, double-blind, placebo-controlled, parallel-group, phase 3 trial	Subcutaneous inclisiran with statins or ezetimibe	781	1.5 mg of subcutaneous injection of inclisiran or matching placebo on Days 1, 90, 270, and 450	66.4	N/A	104.5 mg/dL	422.1 ug/L	Atherosclerotic cardiovascular disease
				Placebo with statins or ezetimibe	780		65.7		104.8 mg/dL	414.9 ug/L	
Ray et al., 2020 [12]	NCT03400800 (ORION-11)	3	Randomized, double-blind, placebo-controlled, parallel-group, phase 3 trial	Subcutaneous inclisiran with statins or ezetimibe	810	1.5 mg of subcutaneous injection of inclisiran or matching placebo on Days 1, 90, 270, and 450	64.8	N/A	107.2 mg/dL	355 ug/L	Atherosclerotic cardiovascular risk equivalent
				Placebo with statins or ezetimibe	807		64.8	N/A	103.7 mg/dL	353 ug/L	

**Table 2:** Safety of inclisiran N/A = not available

Author, Year	Trial name	Most common adverse events (number of events)		
		Adverse events	Inclisiran Group	Placebo Group
Fitzgerald et al., 2014 [6]	N/A	Infusion site hematoma	1	1
							Paresthesia	3	
							Rash	12	4
							Headache	5	2
Fitzgerald et al., 2016 [7]	N/A	Nasopharyngitis	7	N/A
		Diarrhea	4	
		Back pain	5	
		Headache	6	
Fitzgerald et al., 2017 [7,8]	ORION-1	Injection site reaction	19	--
		Myalgias	27	6
Raal et al., 2019 [9]	ORION-2	Paresthesia	1 1	N/A
		Unstable angina	1	
Wright et al., 2020 [10]	ORION-7	Cough	4	N/A
							Nasopharyngitis	1	
							Headache	2	
							Fatigue	2	
Raal et al., 2020 [11]	ORION-9	Nasopharyngitis	28	20
		Influenza	13	21
		Upper respiratory tract infections	16	16
		Back pain	17	10
		Injection site reaction	22	0
Ray et al., 2020 [12]	ORION-10	Death from cardiovascular events	7	5
		Diabetes mellitus	120	108
		Bronchitis	46	30
		Injection site reaction (mild and moderate)	20	7
Ray et al., 2020 [12]	ORION-11	Death from cardiovascular events	9	10
		Injection site reaction	38	4
		Nasopharyngitis	91	90
		Diabetes mellitus	88	94

**Table 3:** Efficacy of inclisiran RI: renal impairment *In these multiple dosing groups studies, the efficacy is written for only the highest and the lowest doses. **This is a single-arm trial.

Author, Year	Trial name	Duration to response measurement (Days)	Administered drugs and dosages	PCSK9 levels mean percentage change from baseline		LDL Cholesterol levels mean percentage change from baseline	
Fitzgerald et al., 2014* [6]	N/A	28	Inclisiran (from 0.015 up to 0.400 mg/kg)	-30.8% at lowest dose		-14.4% for lowest dose	
				-58.6% at highest dose		-36.1% for highest dose	
			Placebo	-8.7%		-24%	
Fitzgerald et al., 2016* [7]	N/A	84	Single-dose inclisiran from 25 mg up to 800 mg	-46.6% at lowest dose		-21.5% at lowest dose	
				-73.1% at highest dose		-43.4% at highest dose	
			Placebo	-0.6%		-10.9%	
			Multiple-dose inclisiran with statins	-79.9% at lowest dose		-45.1% at lowest dose	
				-83.8% at highest dose		-53.2% at highest dose	
			Multiple-dose inclisiran without statins	-75.7% at lowest dose		-52.2% at lowest dose	
				-81.5% at highest dose		-51.7% at highest dose	
			Placebo	+16.9%		-14.2%	
Fitzgerald et al., 2017* [8]	ORION-1	180	Single-dose inclisiran	-47.9% at lowest dose		-27.9% at lowest dose	
				-59.3% at highest dose		-41.9% at highest dose	
			Single-dose placebo	+2.2%		+2.1%	
			Two doses of inclisiran	-53.2% at lowest dose		-35.5% at lowest dose	
				-69.1% at highest dose		-52.6% at highest dose	
			Two doses of placebo	-1.2%		+1.8%	
Raal et al., 2019** [9]	ORION-2	90	Single-dose inclisiran	Patient A: -48.7%		Patient A: +14.3%	
				Patient B: -51.6%		Patient B: -11.7%	
				Patient C: -51.7%		Patient C: -33.1%	
				Patient D: -83.6%		Patient D: -17.5%	
Wright et al., 2020 [10]	ORION-7	60	Single-dose inclisiran	Mild RI	-74.2%	Mild RI	-35.1%
				Moderate RI	-79.8%	Moderate RI	-53.1%
				Severe RI	-67.9%	Severe RI	-49.2%
				Normal RF	-68.1%	Normal RF	-57.6%
Raal et al., 2020 [11]	ORION-9	510	Subcutaneous inclisiran with statins or ezetimibe	-60.7%		-39.7%	
			Placebo with statins or ezetimibe	+17.7%		+8.2%	
Ray et al., 2020 [12]	ORION-10	510	Subcutaneous inclisiran with statins or ezetimibe	-69.8%		-51.3%	
			Placebo with statins or ezetimibe	+13.5%		+1.0%	
Ray et al., 2020 [12]	ORION-11	510	Subcutaneous inclisiran with statins or ezetimibe	-63.6%		-45.8%	
			Placebo with statins or ezetimibe	+15.6%		+4.0%	

Results

The literature search yielded a total of 839 articles. After excluding 291 duplicates, 560 articles were screened based on titles and abstracts. After this first screening, 46 studies were found potentially helpful for our review after excluding 514 items. Full texts of these 46 articles were reviewed, and a further 36 articles were excluded due to one of the following reasons: review papers (n=19), outcomes not measured (n=6), English translation not available (n=2), letters to editors, and other communications (n=9). This study selection and screening process are depicted in Figure 1. Ten articles based on eight clinical trials were included in the final review.

Pre-ORION Trials

Before the ORION trials, two clinical studies were done, and the results were reported in 2014 and 2016, respectively [6,7]. Both were phase 1 randomised single-blinded placebo-controlled trials. In the first trial (n=32, inclisiran group=24, placebo arm= 8), patients in inclisiran group were given inclisiran single infusion (0.015 mg/kg to 0.4 mg/kg). This led to a decrease ranging from 30.8% to 58.5% in levels of PCSK9 from baseline compared to only an 8.7% decrease in the placebo group. Similarly, the decrease in LDL cholesterol levels ranged from 14.4% to 36.1% from baseline compared to a 24% decrease in the placebo group.

In the latter trial (n=69, inclisiran=51, placebo=18), the inclisiran group patients were given either a single dose of 25mg to 800mg inclisiran or multiple doses 125 to 500mg inclisiran once a week to once a month with or without statins. For the single-dose cohort, the decrease in PCSK9 levels ranged from 46.6% to 73.1%. In the multiple doses’ cohorts, this decrease ranged from 79.9% to 83.8% with statins, and 75.1% to 81.5% without statins, compared to an increase in PCSK9 levels by 16.9% in the placebo arm. In comparison, the range of decrease in LDL cholesterol levels in these three cohorts were 21.5% to 43.1%, 45.1% to 53.2%, and 51.7% to 52.2%, respectively.

These trials demonstrated the safety of inclisiran with mild to moderate adverse events, which commonly occurred in both treatment and placebo groups. The most common side effects included injection site reactions, musculoskeletal pain, nasopharyngitis, headache, back pain, and diarrhea. No clinically significant and trending changes were seen on comprehensive metabolic panels, hepatic function tests, cardiac enzymes and inflammatory markers on initiation at six months follow-up. No treatment discontinuations were reported [6,7].

ORION-1

ORION-1 was the first trial of the ORION series to establish the safety and efficacy of inclisiran as a cholesterol-lowering drug. It was a multicenter, double-blind, placebo-controlled trial. The first dose of drug/placebo was given on day 1, and the second dose was given on day 90. In the single-dose group, the percent decrease in PCSK9 levels from baseline ranged from 47.9% to 59.3%, while it increased by 2.2% for the placebo group. For the two doses group, the PCSK9 levels decreased by 75.7% at the lowest dose and 81.5% at the highest dose. In contrast, PCSK9 levels increased by 16.9% for the placebo group. Similarly, the decrease in LDL cholesterol levels ranged from 27.9% to 41.9% and 35.5% to 52.6% in the single and two doses groups.

ORION-1 trial showed the first treatment discontinuations for one patient in the placebo group developing herpes zoster and another developing nasopharyngitis/influenza in the treatment arm. The equivalent occurrence of mild adverse effects, including dizziness, myalgia, headache, fatigue, back pain, hypertension, and diarrhea in individuals getting Inclisiran and placebo, were seen. Injection-site reactions commonly occurred in the inclisiran arm. Transient liver enzyme elevations were seen in both groups with no evidence of hepatocellular injury to warrant drug discontinuation at any point in the trial [8].

ORION-2

The interim results of the ORION-2 trial were presented at the Conference of European Atherosclerotic society in 2019. This trial was intended to profile inclisiran in patients with homozygous familial hypercholesterolemia (HoFH). In the interim results, the responses in four patients were reported. The baseline LDL-C levels in this cohort were 14, 14.2, 15.9, and 4.9 mmol/L. On day 90, the LDL-C increased by 14.3% in the first patient but was decreased by 11.7%, 33.1%, and 17.5%, respectively, in the other patients. But PCSK9 levels decreased in all four patients by 48.7%, 51.6%, 51.7%, and 83.6%, respectively. No safety results have been reported [9]. 

ORION-7

ORION-7 was a phase 1 open-label study designed to test the efficacy of inclisiran in patients with renal insufficiency compared to individuals with normal renal function. All patients were given a single dose of 300 mg Inclisiran. PCSK9 levels decreased by 74.2%, 79.8%, and 67.9% in mild, moderate, and severe renal insufficiency, while it decreased by 68.1% in patients with normal renal insufficiency. Similarly, the LDL-C decreased by 35.1%, 53.1%, and 49.2% in patients with mild, moderate, and severe renal insufficiency and decreased by 57.6% in the normal renal function group [10].

Inclisiran was also well-tolerated in groups with normal kidney function and those with mild, moderate, and severe renal injury in ORION-7. No requirement for dose adjustment in patients with renal impairment was warranted for the same or more reduction in LDL levels based on the data reported [10].

ORION-9

ORION-9 was a phase 3, double-blind, placebo-controlled trial to assess the efficacy of inclisiran in patients with heterozygous familial hypercholesterolemia. The treatment group (n=242) was administered 300 mg of inclisiran on Days 1, 90, 270, and 450, while the control group (n=240) was given a placebo. The mean PCSK9 levels decreased by 60.7% in the inclisiran group on 510th day follow up, but it was increased by 17.7% in the placebo group. Similarly, the mean LDL cholesterol decreased by 39.7% in the inclisiran group but was increased by 8.2% in the control group [11].

The commonly reported adverse events in the inclisiran arm were nasopharyngitis, back pain, and upper respiratory tract infections. However, there was no statistically significant difference in these events' risk ratios in the treatment and control groups. The only significant side effect in the treatment group was injection site reaction (n=22), and none of those reactions was graded as severe or persistent [11].

ORION-10 and -11

ORION-10 (n= 1561) and ORION-11 (n= 1617) were two similarly designed placebo-controlled trials to assess the efficacy of inclisiran in a larger population. The patients included in these two trials had atherosclerotic cardiovascular disease or were at high risk of it. The results of both these phase 3 trials are published in 2020. The inclisiran group patients were given 1.5 ml of subcutaneous injection of inclisiran on Days 1, 90, 270, and 450. The mean PCSK9 levels at day 510 were decreased by 69.8% and 63.6% in treatment groups of ORION-10 and ORION-11, respectively. But it increased by 13.5% and 15.6% in the control group of both these trials, respectively. Moreover, the LDL cholesterol also decreased by 51.3% and 45.8% in the inclisiran group of ORION-10 and -11, respectively, but it increased by 1% and 4% in control groups of these trials. 

As with ORION-9, the only statistically significant adverse event occurring in the treatment arm were injection site reactions in both trials. However, all of them were graded as mild or moderate [12].

Discussion

The relationship between low levels of LDL-C and improved cardiovascular outcomes is well established. Higher LDL levels were associated with a higher ASCVD score with a consequent more significant risk of forthcoming adverse cardiovascular events [13]. Mortality rates were improved even in younger populations with higher LDL levels. Lipid-lowering drugs were studied to reduce mortality, and statins were and are one of the foremost lipid-lowering drugs [14]. They are also used for their anti-inflammatory effect in patients with recent stroke and myocardial infarction to stabilise atherosclerotic plaque [15]. The reduction in adverse events and mortality rate was even seen in patients with baseline low LDL levels treated with LDL lowering pharmacological therapy.

The mechanism of action of inclisiran is based on PCSK9 inhibition, which dictates the LDL levels circulating in serum by enzyme-mediated degradation of LDL receptors [16]. This mechanism's idea of pharmacological antagonism, giving birth to inclisiran and PSCK9 antibodies, originated from studying the gain of function mutation for the PCSK9 gene in inherently predisposed individuals. They were seen usually clustered in families. The gain of function of the PCSK gene, which encodes NARC-1 (neural apoptosis regulated convertase) contributing to cholesterol homeostasis, is studied as the basis for familial hypercholesterolemia. This entity is a known atherosclerotic risk factor predisposing individuals to adverse cardiovascular events and premature CAD [17-19].

Similarly, the opposite phenomenon with a mutation of this gene with a diminution of function was observed in specific population groups with lower LDL levels and better cardiovascular outcomes and reduced mortality [3, 20-25]. A reduced incidence of myocardial infarction and even aortic valve stenosis was seen in these patients with lower PCSK9 expression [26]. A study was carried out in Japan to study the genetic mutations in patients, which reiterated the same results [27]. Another large cohort study is on its way to establish the association of PCSK9 mutations with LDL levels and consequent cardiac event(s) [28].

The efficacy of inclisiran in reducing LDL levels has been evident in all the clinical trials included in our study. The initial phase 1 studies were randomised and single-blind studies; reduction of LDL-C was seen in both single-dose and multiple-dose studies [6,7]. Subsequent trials were multi-centric, double-blind, placebo-controlled studies. They demonstrated the same results, albeit with more statistical significance due to larger cohorts including a healthier population and the patients with mild, moderate to severe renal insufficiency, known atherosclerotic disease, or at risk of adverse cardiovascular events and in patients with hypercholesterolemia. There was one, possibly, aberrant reporting of increased LDL in one patient in ORION-2 [9], but the PCSK9 levels were lowered in all participants. That patient’s LDL levels were previously resistant to anti-PCSK9 antibodies. It may suggest a guideline for avoiding the use of inclisiran and similar drugs in the future in the specific patient cohort. Inclisiran has the edge over prior lipid-lowering medications due to a large margin of efficacy and convenient dosing intervals [8,11,12].

The majority of the adverse effects in all trials included in our study were classified as mild to moderate and were manageable with no long-term side effects (Table 2). Safety and efficacy in initial tests were questioned due to the smaller sample size. A large number of the patients from the sample size in ORION=1 belonged to the Caucasian group [8]. The efficacy could not be well demonstrated for the multi-dose phases as subsequent doses of inclisiran if administered within the first dose's maximal pharmacodynamics activity, which could lead to apparently moderate incremental effects of increasing amounts on the levels of PCSK9 and LDL cholesterol.

The number of participants was then increased in randomised subsequent trials to assess safety profiles and pharmacodynamics of inclisiran better. The skin site reactions, including rash, were reported as mild and transient and occurred with similar frequency in both groups (Table 2). A possible underlying ascertainment bias was reported in the background due to over-reporting of this side effect from one particular clinical site in pre-ORION trials [6,7]. No long-term skin changes were observed. Considering the premedication if administered and proper skin reaction classification using a predefined criterion with appropriate follow-up can help reduce this adverse effect.

Two fatalities were reported later in the ORION-1 trial. A patient assigned to the single-dose 500-mg inclisiran group with a long-standing peripheral vascular disease and recurrent anginas who underwent a cardiac arrest died on Day 104. Another man in the two-dose 200-mg inclisiran group died on day 198 in trial with a surgical repair of thoracic aortic aneurysm and subsequent complication by fistula and sepsis after participation in the trial. The attribution of any of these fatal outcomes to inclisiran has been difficult to explain due to multiple comorbidities and unforeseeable health calamities, which had no basis for a possible drug-related reaction. Platelet levels and basic metabolic panels were stable, and no other abnormalities were documented [8].

Elevated gamma-glutamyl transferase (GGT) was reported in a patient with concomitant statin therapy, which improved on discontinuation and recurred on initiation, classifying this elevation as most likely related to statin therapy. Initial studies had a meagre number of patients already taking statins, and intake of additional lipid-lowering drugs like ezetimibe or others was not screened [6,7]. Subsequent inclusion of patients with known atherosclerotic disease or at high risk for it with randomisation ensured an appropriate representation of patients already receiving statins, and no possible drug-drug interactions were reported. ORION-9 trial showed low-titer antidrug antibodies consistent with assay-testing characteristics and not considered to be due to treatment with inclisiran [9]. The presence of these antibodies was transient and not associated with changes in any pharmacologic or clinical measurements. 

The long-term results of phase 3 LDL-C lowering studies (ORION trials 9, 10, 11) and results of ongoing clinical trials like ORION-4 will provide us with more data to comment on the renal and cardiovascular safety of inclisiran. The efficacy of injecting inclisiran twice is reported to be greater than one-time dosing in earlier clinical studies. The data from future trials will help us take a proper prophylactic and preventive approach in these patients while prescribing inclisiran or recruiting patients in subsequent trials. Previously used lipid-lowering drugs have an established efficacy but an undesirable side effect profile, including elevated liver enzymes, drug interactions, and rhabdomyolysis [28]. Paradoxically, some studies showed that the PCSK9 gene levels were upregulated in patients taking statins as lipid-lowering agents, but more studies are needed to prove this causation [29]. The frequency of administration of inclisiran is convenient (twice yearly compared to every four to six weeks for the monoclonal antibodies). The final pricing for inclisiran has not been commented upon but is fairly priced compared to the monoclonal antibodies. Inclisiran can emerge as a superior substitute for lowering both serum LDL levels and subsequent risks of adverse cardiovascular events.

## Conclusions

In light of the recent randomized placebo-controlled trials, inclisiran has proven efficacious in a wide variety of patient populations. Inclisiran has shown a promising safety profile. Severe adverse events and fatalities noted in trials have not been related to inclisiran. The results of the ongoing clinical trials will help us to corroborate these findings further. They would open vistas for the potential application of inclisiran in suitable and responsive patient populations.
